# A Randomized Trial of Inpatient and Home-Based Maternal Oral Hydration Therapy in Isolated Oligohydramnios and Its Effect on Amniotic Fluid Index and Perinatal Outcome

**DOI:** 10.7759/cureus.41326

**Published:** 2023-07-03

**Authors:** Monika Anant, Suhagini Murmu, Swati Priya

**Affiliations:** 1 Obstetrics and Gynecology, All India Institute of Medical Sciences, Patna, Patna, IND; 2 Obstetrics and Gynecology, All India Institute of Medical Sciences, Deoghar, Deoghar, IND

**Keywords:** oral hydration therapy, isolated oligohydramnios, birth weight, apgar, amniotic fluid

## Abstract

Background

Isolated oligohydramnios, without any known fetal/maternal abnormality, may be associated with insufficient oral intake (such as water, glucose, and rehydration therapy). Therefore, the present study was conducted to assess the improvement following maternal hydration.

Method

A total of 50 cases of isolated oligohydramnios (other high-risk pregnancy conditions not present) were included in the study (25 in each group). Patients were encouraged for an additional 2 liters of oral rehydration solution intake daily along with regular diet. The fluid intake was unsupervised in the home group and supervised in the hospital group. Serial amniotic fluid index (AFI) measurements and fetal monitoring were performed. Birth weight and APGAR scores were recorded, and data were analyzed.

Results

The two groups were comparable in terms of demographics and baseline laboratory findings. AFI significantly improved in the hospital group compared to the home group (p-value: <0.001). Birth weight, placental weight, and APGAR scores were also significantly better in the hospital group than in the home group.

Conclusion

Maternal oral hydration therapy improves the amniotic fluid volume and subsequently improves the perinatal outcome. Due to poor compliance with home-based treatment, institution of supervised hydration therapy is recommended.

## Introduction

Amniotic fluid is the clear, slightly yellowish fluid surrounding the fetus. It is contained in the amniotic sac. It is predominantly produced by fetal secretions, including urine, oral, nasal, tracheal, and pulmonary secretions [1-2]. Removal is predominantly by fetal swallowing and absorption through the membranes [3]. The amount of amniotic fluid in the gestational sac is the result of the dynamic equilibrium of the aforementioned processes.

Amniotic fluid serves many purposes. It acts as a vital source of nutrients and growth factors for the growing fetus, provides mechanical and cushioning support to the fetus, provides a protective barrier, and acts as the innate immune system [4-5]. It also plays a vital role in aiding the prenatal diagnosis of structural and chromosomal abnormalities, prediction of premature rupture of membranes (PROM), and assessment of fetal lung maturity.

Clearly, amniotic fluid is essential for the normal growth and development of the fetus. Amniotic fluid volume is determined sonographically by amniotic fluid index (AFI). Oligohydramnios is defined as AFI of less than 5 cm or single largest pocket of less than 2 cm in either the horizontal or the vertical plane [6-7]. It complicates 0.5% to 5% of all pregnancies [8].

Understandably, any condition interfering with production and/or absorption of the amniotic fluid can lead to serious disturbances. These include conditions such as fetal genitourinary abnormalities and fetal gastrointestinal abnormalities. Maternal conditions such as hypertension, diabetes, insufficient oral fluid intake, and post-term pregnancy are other established etiologies. Maternal hormonal imbalance can also lead to changes in fetal urine osmolality and can adversely affect the amniotic fluid volume.

Oligohydramnios is associated with adverse pregnancy outcomes with increase in maternal and perinatal morbidity and mortality. These include poor progress of labor and increase in cesarean section rates, preterm delivery, pulmonary hypoplasia, low APGAR scores, meconium passage, structural abnormalities, and neonatal intensive care unit (NICU) admissions [9-10].

Despite the varied etiology of oligohydramnios, in some cases it is seen in appropriate-for-gestational age fetus without any fetal or maternal disease. These cases are termed as isolated oligohydramnios (IO) [11]. IO is generally associated with insufficient maternal fluid intake, and, consequently, the prognosis can be improved by increasing the maternal oral intake of fluids [12-14]. However, the studies comparing the improvement in the home and hospital settings are scarce, especially in Indian demographics. Therefore, this study was conducted to assess the improvement in AFI and perinatal outcome following maternal oral hydration therapy in home and hospital settings.

## Materials and methods

This prospective, interventional, randomized controlled trial was conducted in the Department of Obstetrics and Gynecology, All India Institute of Medical Sciences, Patna. It was conducted after obtaining permission from the Institutional Ethics Committee. Antenatal patients who were diagnosed to have oligohydramnios (AFI less than 5 cm, AFI of 5 cm to 8 cm) in the third trimester (28 completed weeks to 40 weeks) and who met the inclusion and exclusion criteria as below were included in the study.

Ultrasound-confirmed IO is defined as severe oligohydramnios (when AFI is less than 5 cm) and moderate oligohydramnios (when AFI is 5 cm to 8 cm). All non-high-risk, singleton pregnancies with oligohydramnios were included.

All pregnant women with intrauterine growth retardation (IUGR), maternal hypertension, diabetes, chromosomal/structural abnormalities in the fetus, premature rupture of membranes, multiple pregnancies, evidence of fetal infection, scarred uterus (previous caesarean section, myomectomy, hysterotomy), and malpresentation were excluded. Patients refusing to consent for the study were also excluded.

As prior literature was not available for the study, values for real and acceptable difference were assumed. Taking a significance level at change of AFI as >2 cm, change in AFI between both arms was calculated taking confidence interval as 95% in a total of 50 pregnant women included in this pilot study. A written informed consent was obtained from all the patients. Antenatal women enrolled in the study were assigned one of the two groups, namely, home group and hospital group, of 25 each. Random allocation was done by an analyst using software-generated random number sequence. Demographics and baseline physical examination findings were recorded. Basic laboratory investigations were conducted. All patients underwent ultrasound on day 1. Measurement of liquor pockets (anechoic) in all four quadrants of the abdomen was recorded. The sum of all in centimeters was recorded as AFI. The baseline AFI at day 1 was recorded for all the patients.

The patients included in the home group received proper advice regarding extra fluid intake of 2 liters of oral rehydration solution (ORS) with their normal diet and fluid intake and proper rest (minimum 2 hours in daytime and 8 hours sleep at night in the left lateral position rest) and sent home after a non-stress test (NST) and biophysical profile (BPP) recording. NST tracing was recorded in patients with period of gestation of more than 32 weeks (after eight months of pregnancy). NST machine probe was securely put over the patient’s abdomen and fetal heart tracing obtained for 20 minutes. BPP was done by ultrasound examination of fetal body movement, fetal muscle tone, breathing movement, heart rate, and AFI. They were called after seven days for a repeat evaluation of AFI. The patients included in the hospital group were admitted in the hospital for a week and given supervised extra fluid intake orally of 2 liters of ORS and proper rest and normal diet. After a week, AFI measurements were repeated for both the groups. Patients in the hospital group were then discharged. Both the groups were advised to follow up weekly for the assessment of AFI and fetal monitoring. They were monitored as outpatients till delivery. Weekly NST and BPP screenings were conducted.

For patients with borderline AFI (5 cm to 8 cm), surveillance was done with weekly ultrasound scan for growth, AFI, and Doppler till delivery. Ultrasound Doppler of uterine arteries was conducted. The S/D ratio (systolic peak/diastolic end) and PI (pulsatility index) was recorded in all patients. If AFI was less than 5 cm at 37 weeks, either induction of labor was done or surveillance was continued after counselling about risks and benefits on an individualized basis.

For patients with AFI less than 5 cm and preterm, surveillance was done with twice weekly cardiotocography (CTG), weekly scans for AFI, and Doppler till term or until AFI returned back to normal. Corticosteroids were administered for fetal lung maturity.

The details of mode of delivery and birth weight were recorded. Perinatal outcomes of both the groups were assessed, and APGAR scores at 1 and 5 minutes and requirement for admission to a NICU were noted.

**Figure 1:**
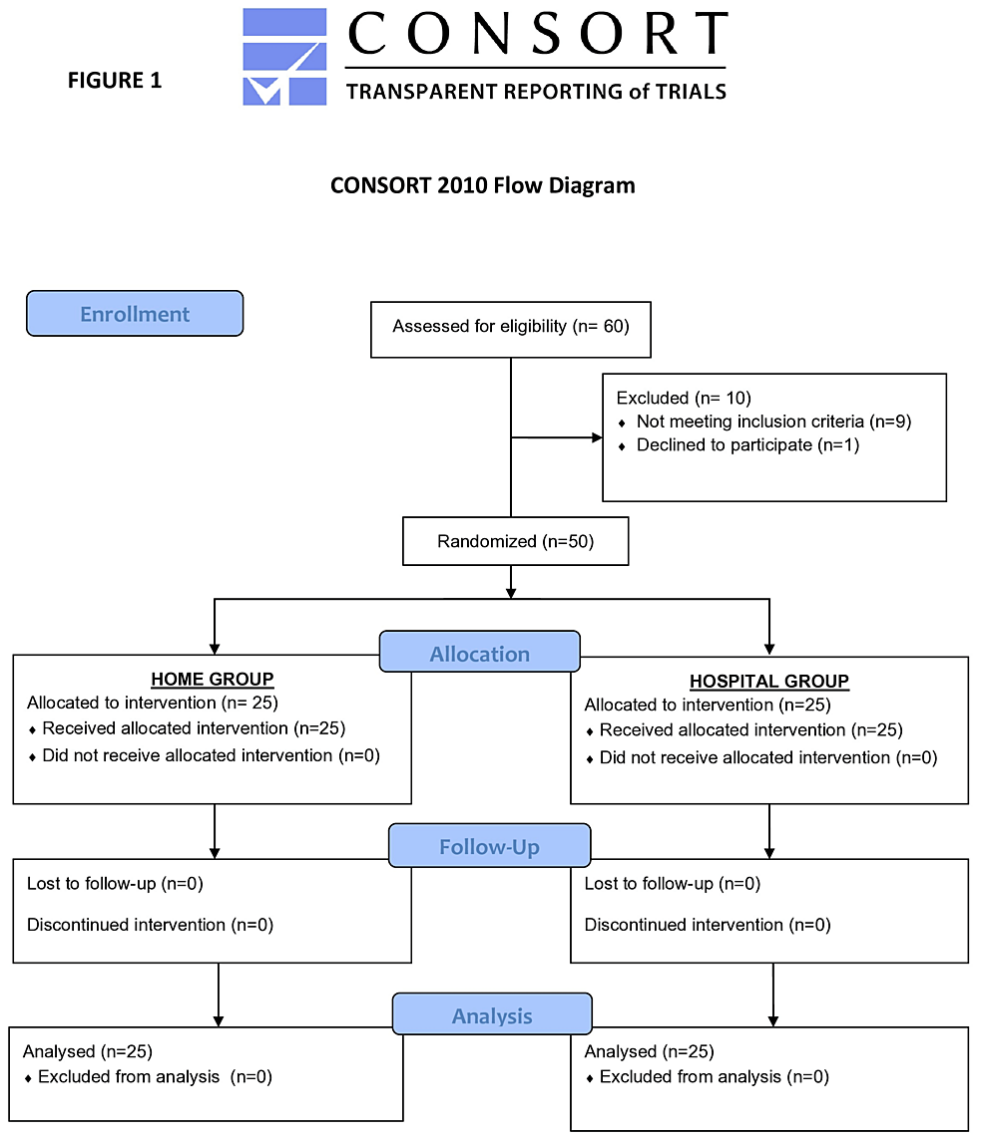
Methodology (CONSORT 2010 flow diagram)

Analysis was carried out using SPSS (IBM Corp., Armonk, NY). Quantitative parameters were described in terms of mean and standard deviation. The P-value was calculated by “unpaired t test” for quantitative data. A p-value of less than 0.05 was considered statistically significant (Figure 1).

## Results

The mean age of the population treated at home was 23.04 ± 3.70 years, and of those treated at the hospital, it was 25.24 ± 4.04 years (p-value: 0.051). The gestational age of the patients was almost similar in both the groups (home: 274.80 ± 7.55 days, hospital: 274.24 ± 10.40 days; p-value: 0.829). The baseline mean blood pressure was same in both the groups (Table 1).

**Table 1:** Distribution of the baseline blood pressure according to the type of intervention

Parameter	Home	Hospital	P-value
Systolic blood pressure (mmHg)	113.64 ± 10.13	116.24 ± 8.63	0.333
Diastolic blood pressure (mmHg)	77.32 ± 7.44	75.80 ± 5.46	0.414

The laboratory findings were also similar in both the groups (Table 2).

**Table 2:** Distribution of the baseline laboratory investigations according to the type of intervention

Parameter	Home	Hospital	P-value
Hemoglobin (g/dL)	11.27 ± 1.61	12.02 ± 2.09	0.161
Glucose following glucose tolerance test (mg/dL)	109.04 ± 20.67	112.27 ± 18.93	0.567
Thyroid-stimulating hormone (mIU/L)	2.31 ± 1.01	2.17 ± 0.98	0.605

The mean AFI at day 1 was similar in both the groups, while AFI at day 7 was significantly higher in the hospital group (p-value: 0.001) (Table 3).

**Table 3:** Distribution of the AFI before and after treatment according to the type of intervention *Value is statistically significant as p-value is <0.05 AFI, amniotic fluid index

Parameter	Home	Hospital	P-value
AFI at day 1	7.00 ± 1.26	6.82 ± 1.44	0.639
AFI at day 7	6.76 ± 1.79	8.70 ± 2.01	0.001*

The birth weight and placental weight were also more in the hospital group (Table 4).

**Table 4:** Distribution of the birth weight and placental weight according to the type of intervention *Value is statistically significant as p-value is <0.05

Parameter	Home	Hospital	P-value
Birth weight (kg)	2.78 ± 0.22	3.03 ± 0.35	0.004*
Placental weight (gm)	466.40 ± 29.03	499.64 ± 27.64	<0.001*

The APGAR score at 5 minutes was higher in the hospital group (Table 5).

**Table 5:** Distribution of the APGAR score according to the type of intervention *Value statistically significant as p-value is <0.05 APGAR is appearance, pulse, grimace, activity, and respiration

Parameter	Home	Hospital	P-value
APGAR at 1 minute	8.20 ± 0.91	8.68 ± 1.07	0.094
APGAR at 5 minutes	9.64 ± 0.49	10.00 ± 0.00	0.001*

## Discussion

In our study, the two groups were similar in terms of demographic variables (age, socioeconomic status) and baseline laboratory findings (hemoglobin, oral glucose tolerance test, and thyroid-stimulating hormone).

The assessment of AFI at day 1 was similar in the two groups. After hydration intervention of the groups, the AFI at day 7 was lower in the group treated at home as compared to those treated at hospital. A similar case-control study was conducted by Chauahan et al. [13] with 50 patients in each group. Cases were advised 2 liters of water over 6 hours for seven days, in addition to their daily fluid intake, while no hydration intervention was instituted in the control group. They observed a significant increase in AFI from day 0 to day 7 in the case group. Intravenous hydration has also been studied with a resultant increase in liquor volume by Umber and Chohan [14]. Other studies also confirm these findings [15-17].

Different quantities of oral fluid intake affecting AFI has also been studied by Patrelli et al. [18]. They had subgroups with intake of 1,500 mL/day and 2,500 mL/day of home oral hydration therapy. They observed that the differences among the subgroups were significant even at the time of birth. Results were corroborated by another study [19]. Our study, however, has not assessed different rates of fluid intake with resultant AFI.

Fetal urine production rate in the near-term fetus after acute maternal hydration has been reported by Oosterhof et al. [20]. The behavioral state of the fetus (heart rate, eye movements, and body movements) was also included for the study. The hourly fetal urine production rate increased significantly after maternal rehydration as compared to that after 4 hours of fluid deprivation, concluding that the increased hourly fetal urine production leads from either acute changes in maternal fluid volume or maternal plasma osmolality or both.

It has been hypothesized that improvement in maternal hydration status leads to a decrease in plasma osmolality and consequently increased uteroplacental perfusion. This ultimately leads to an increase in fetal urine production, thereby increasing the amniotic fluid volume.

When assessed for outcome parameters, it was observed that the birth weight of the babies in the patients treated at the hospital was significantly more than that in the patients treated at home. Similar trends were observed for the placental weight also. Though the APGAR score of the babies at 1 minute was similar in the two groups, a significant difference was noted at APGAR of 5 minutes with favorable scores of the babies of mothers treated to oral hydration in the hospital as compared to those treated at home.

Pregnancy continued for a significantly longer duration in the case group in a study by Chauahan et al. [13]. The birth weight and APGAR scores were also significantly better in the hydration group than the control group. Increase in birth weight and APGAR scores with improvement of oligohydramnios was observed in other studies as well [19,21-22]. The effect of increasing birth weight and improved fetal outcomes maybe attributed to the increased uteroplacental perfusion.

Traditionally, oligohydramnios has been associated with increased perinatal morbidity and mortality due to the compression of the umbilical cord resulting in intermittently decreased uteroplacental flow [23-24]. Understandably, decreased fetal blood supply has an adverse effect on normal fetal development resulting in low birth weight and poor APGAR scores. Thus, improvement in the AFI improves the perinatal outcome, as observed in the present study.

Comparison of the effects of home versus hospital management clearly favored hydration therapy at hospital with better outcomes in our study. It is obvious that decreased compliance in Indian patients managed on home-based care is due to unsupervised treatment regimen. Poor compliance with home-based treatment, especially adherence to iron and folic acid supplementation, has been reported in many studies [25-29]. Various reasons cited are nuclear family, forgetfulness, side effects, ignorance, inadequate counselling, and poor literacy levels. Therefore, regular antenatal check-ups are recommended for early detection of oligohydramnios along with advice of increased oral fluid intake, preferably by hospital admission.

The present single-center study was limited by the outpatient department attendance of the patients. Therefore, the results may not be generalized.

## Conclusions

It can be effectively concluded from the present study that oral hydration therapy significantly improves the amniotic fluid volume. The resultant improvement in amniotic fluid volume translates to improved perinatal outcome in terms of birth weight and APGAR scores. Furthermore, institution of supervised hydration therapy is recommended due to poor compliance in developing countries, preferably by hospital admission (if required).
